# Plasma Krebs Cycle Intermediates in Nonalcoholic Fatty Liver Disease

**DOI:** 10.3390/jcm9020314

**Published:** 2020-01-22

**Authors:** Yana Sandlers, Rohan R. Shah, Ryan W. Pearce, Jaividhya Dasarathy, Arthur J. McCullough, Srinivasan Dasarathy

**Affiliations:** 1 Department of Chemistry, Cleveland State University, Cleveland, OH 44115, USA; 2 MetroHealth Medical Center, Case Western Reserve University, Cleveland, OH 44139, USA; 3 Department of Inflammation and Immunity, Lerner Research Institute, Cleveland Clinic, Cleveland, OH 44115, USA; 4 Department of Gastroenterology and Hepatology, Lerner Research Institute, Cleveland Clinic, Cleveland, OH 44115, USA

**Keywords:** plasma Krebs cycle intermediates, nonalcoholic fatty liver disease (NAFLD), mitochondrial dysfunction

## Abstract

Nonalcoholic liver disease (NAFLD) is manifested with a wide spectrum of clinical symptoms and is closely associated with the metabolic syndrome, inflammation, and mitochondrial dysfunction. Although the mechanism of mitochondrial dysfunction in NAFLD is still not fully elucidated, multiple studies have demonstrated evidence of molecular, biochemical, and biophysical mitochondrial abnormalities in NAFLD. Given the association between NAFLD and mitochondrial dysfunction, the aim of this study is to analyze circulating levels of Krebs cycle intermediates in a cohort of NAFLD-affected individuals and matching healthy controls and to correlate our findings with the liver function metrics. Standard serum biochemistry and Krebs cycle intermediates were analyzed in NAFLD (*n* = 22) and matched control (*n* = 67) cohorts. Circulating levels of isocitrate and citrate were significantly (*p* < 0.05) elevated in the NAFLD cohort of patients. The area under the curve (AUROC) for these two metabolites exhibited a moderate clinical utility. Correlations between plasma Krebs cycle intermediates and standard clinical plasma metrics were explored by Pearson’s correlation coefficient. The data obtained for plasma Krebs cycle intermediates suggest pathophysiological insights that link mitochondrial dysfunction with NAFLD. Our findings reveal that plasma isocitrate and citrate can discriminate between normal and NAFLD cohorts and can be utilized as noninvasive markers of mitochondrial dysfunction in NAFLD. Future studies with large populations at different NAFLD stages are warranted.

## 1. Introduction

Nonalcoholic fatty liver disease (NAFLD) is the most common liver disorder in the United States, potentially leading to fibrosis, cirrhosis, and hepatocellular carcinoma progress [1,2,3,4]. NAFLD is manifested with a wide spectrum of clinical symptoms while disease pathogenesis is described through a “multiple-hit model.” The model takes into consideration multiple factors that may contribute to the disease progress and staging [5]. Indeed, NAFLD is closely associated with metabolic syndrome, inflammation, and mitochondrial dysfunction [4,6,7,8]. Although the mechanism of mitochondrial dysfunction in NAFLD is still not fully elucidated, multiple studies have demonstrated evidence of molecular, biochemical, and biophysical mitochondrial abnormalities in NAFLD. Mitochondria play a very significant role in hepatic cellular metabolism. Indeed, NAFLD-affected individuals exhibit morphological changes in liver mitochondria and a decrease in ATP production [9]. Decreased expression of mtDNA-encoded polypeptides and low activity of complexes I, III, and V were also found in nonalcoholic steatohepatitis (NASH) patients [10]. Studies in animal models demonstrate oxidative stress, decreased activity of OXPHOS proteins, high levels of circulating TNF-α, and accumulation of hepatic cardiolipin and ubiquinone [9,11]. Regardless of the mechanism involved, these data provide compelling evidence that mitochondrial dysfunction contributes to the NAFLD phenotype. In the setting of impaired mitochondrial function, Krebs cycle involvement in NAFLD pathogenesis is expected. The Krebs cycle is a central mitochondrial pathway that serves as a metabolic hub for aerobic respiration, glycolysis, lipogenesis, gluconeogenesis, and amino acid synthesis. For every turn of the cycle, there is a simultaneous reduction of NAD^+^ and ubiquinone. Subsequent oxidation of NADH and FADH_2_, followed by ADP phosphorylation, leads to ATP synthesis. Disruption of the Krebs cycle may lead to a wide range of metabolic disturbances and clinical symptoms. Given the association between NAFLD and mitochondrial dysfunction, the aim of this study is to analyze circulating levels of Krebs cycle intermediates in a cohort of NAFLD-affected individuals and matching healthy controls, and correlate our findings to the liver function clinical metrics.

## 2. Experimental Section

### 2.1. Subjects

NAFLD (*n* = 22) and matched control cohorts (*n* = 67) included both genders in the age range 23–67 years old. The diagnosis of NAFLD was made on clinical and ultrasound evidence and by excluding other causes of abnormal liver function tests. The sonographic findings have been validated in the past [12]. Blood samples were drawn presumably after an overnight fast, although later plasma glucose analysis revealed that not all subjects were compliant with the fasting protocol. Informed consent was obtained from all subjects and the procedures were conducted in compliance with the Institutional Review Board at Metro Health Medical Center. All plasma sample were analyzed for liver transaminases (alanine aminotransferase (ALT) and aspartate aminotransferase (AST), alkaline phosphatase (ALP), blood urea nitrogen (BUN), bilirubin, albumin, creatinine, glucose, HbA1C, triglycerides (TG), total cholesterol, high-density lipoprotein cholesterol (HDL-C), and inflammatory markers.

### 2.2. Sample Preparation for Krebs Cycle Intermediates

A total of 350 µL of plasma was spiked with 50 µL of a 0.2 mM mixture of tricarballylic acid, ^13^C_4_-malate (Millipore Sigma, Burlington, MA, USA), d_6_-succinate (Millipore Sigma), d_6_-α-ketoglutarate (Millipore Sigma), and 2 mM of ^13^C_6_-citrate (Millipore Sigma) followed by the addition of 25 μL of ^13^C_-4_-fumarate (Millipore Sigma) in ethanol (0.02 mM), then 1 mL of 1 N HCl in a saturated NaCl mixture and 1 mL of ethyl acetate were added. Tubes were vortexed, then rocked for ten more minutes. The slurry was centrifuged at 1000 rpm for 10 min, then the upper organic phase was carefully transferred to the clean reaction tube. Ethyl acetate extraction was repeated one more time, and organic extracts were combined into one tube. Samples were completely dried under a nitrogen stream at room temperature and incubated with 40 µL of metoxyamine in pyridine (20 mg/mL) at 80 °C for 1 h. Then tubes were cooled to the room temperature and 60 µL of bis-trimethylsilytrifluoroacetamide (BSTFA)/1% trimethylchlorosilane (Millipore Sigma) was added following incubation at 70 °C for 45 min. Samples were transferred to the gas chromatography mass spectrometer (GCMS) vials.

### 2.3. Gas Chromatography-mass Spectrometry (GCMS) Analysis

GC-MS analysis was performed with Agilent 5977. A mass spectrometer coupled to a 7890 B gas chromatograph fitted with a 7693 autosampler and a DB-5ms column (Agilent, Santa Clara, CA, USA). The GC-MS was operated as electron impact (EI)/single ion monitoring (SIM) mode. Target ions and retention times can be found in Supplemental Materials. The temperature program was as follows: 80 °C hold for 2 min, increase 15 °C/min up to 305 °C and hold for 3 min. Calibrations curves with at least six points were obtained by plotting the metabolite/internal standard peak ratio versus the metabolite concentrations in spiked plasma followed by linear regression analysis. The criteria for acceptance was set as a correlation coefficient r^2^ > 0.99. Carryover was examined by extracting spiked plasma samples with a high level of analytes followed by GC-MS runs of these samples and blanks. The coefficients of inter- and intraday variation and accuracy of the spiked samples were within acceptable limits (CV ≤ 20%).

#### Aconitate and Isocitrate Quantification

Since no commercially available stable isotope-labeled standards for aconitate and isocitrate were found, tricarballylic acid (Supplemental material, Figure S2) was used as an internal standard for these metabolites.

## 3. Results

Serum biochemistry was assessed including glucose, HbA1c, plasma creatinine, BUN, bilirubin, albumin, triglycerides, total cholesterol, HDL-C, ALT, and AST, TNF-α and leptin. Table 1 summarizes the mean standard blood clinical metrics obtained for NAFLD (*n* = 22) and matching controls (*n* = 67). Some of the study participants had non fasting glucose levels, so Table 2 summarizes clinical metrics for samples with plasma glucose ≤100 mg/dL. Non fasting glucose samples were excluded from Table 2 regardless of the HbA1c levels.

HbA1c—although the average value for the glycated hemoglobin (HbA1c) in the NAFLD group didn’t exceed the 6.5% cut-off, this value indicates prediabetic conditions. Overall, 32% (7 out of 22) of the NAFLD cohort had HbA1c ≥ 6.5%.

Albumin—decreased albumin levels are associated with cirrhosis, septal, intensive fibrosis, and steatohepatitis. Analysis of the entire cohort including samples with the non fasting glucose revealed that the average albumin level in affected individuals was decreased (3.8 ± 0.4 vs. 4.1 ± 0.2, respectively), *p* < 0.05 (Table 1). After the exclusion of samples with non fasting glucose levels, no difference was found in albumin values (Table 2).

Transaminases—alanine aminotransferase (ALT) activity, a marker of the hepatic damage, was significantly elevated (*p* < 0.05) regardless of the fasting status. The difference in aspartate aminotransferase (AST) activity between the normal and affected cohort became more evident in the group with glucose <100 mg/dL cut-off. The AST/ALT ratio, which is often used as an independent predictive factor for advanced hepatic fibrosis [13], was not significantly different between the two groups (Table 1). It is important to note that fifty percent of the NAFLD population can present with normal AST and ALT levels [14,15].

Bilirubin—although bilirubin levels were reported to be inversely associated with NAFLD [16], we didn’t observe a statistically significant difference in our cohorts. 

Plasma creatinine, a common marker in cirrhosis and chronic kidney disease, also was not significantly different.

Increased triglycerides (TG) and decreased high-density lipoprotein cholesterol (HDL-C) are associated with metabolic syndrome [17]. It is interesting to note that the NAFLD cohort had elevated triglycerides but no difference in HDL-C level. Despite this, the triglycerides/high-density lipoprotein cholesterol ratio, reported in association with insulin resistance, was elevated in the NAFLD group.

Body mass index (BMI) is associated with fatty liver risk [18]. In this regard, we found a statistically significant BMI increase in the NAFLD cohort (Table 1), which became less apparent when only samples with glucose cut-off <100 mg/dL were included for the analysis (Table 2). 

Hepatic steatosis index (HSI) was calculated using demographic and standard plasma metrics (Figure S4, Supplemental materials) [19]. In accordance to a study by Lee et al., HSI correlates significantly with fatty liver grade with a screening cut-off of >36.0 and likely reflects the presence of nonalcoholic steatohepatitis [19].

The Krebs cycle has a central role in liver metabolism, connecting hepatic fatty acid and glucose-related pathways. Abnormal cellular [20] and circulating [21] Krebs cycle intermediates are closely associated with mitochondrial dysfunction as was demonstrated in numerous studies [22,23,24,25]. Given the decreased activity in respiratory chain complexes in NAFLD [4], perturbations in cellular concentrations of Krebs cycle intermediates are expected.

**Figure 1:**
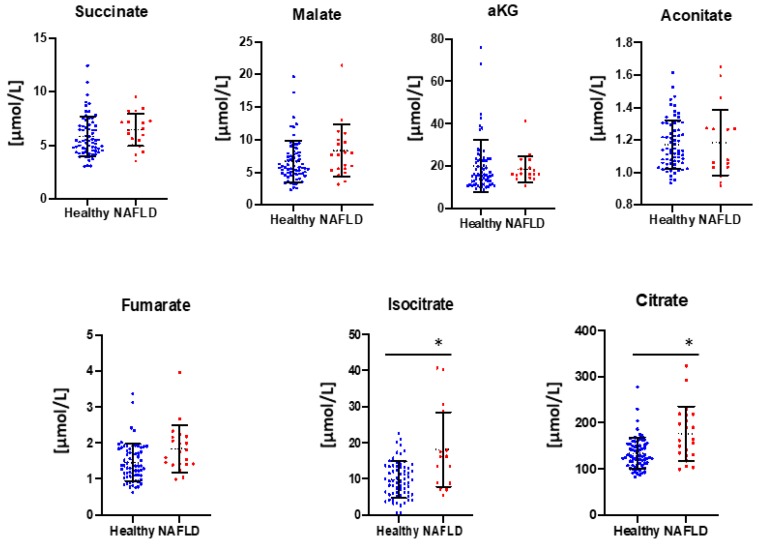
Plasma Krebs cycle intermediates levels by GCMS. Nonalcoholic fatty liver disease (NAFLD) (*n* = 22) and controls (*n* = 67). Statistical analysis performed by Prism 8 (GraphPad). * *p* < 0.05.

Seven circulating Krebs cycle intermediates were analyzed including citrate, isocitrate, aconitate, α-ketoglutarate, succinate, fumarate, and malate. Oxaloacetate is not chemically stable and hence was omitted from the analyses. Succinyl-CoA is a cellular metabolite and consequently is not abundant in human plasma. Statistically significant increases were detected in circulating levels of citrate and isocitrate regardless of the plasma glucose levels (Figure 1).

**Figure 2:**
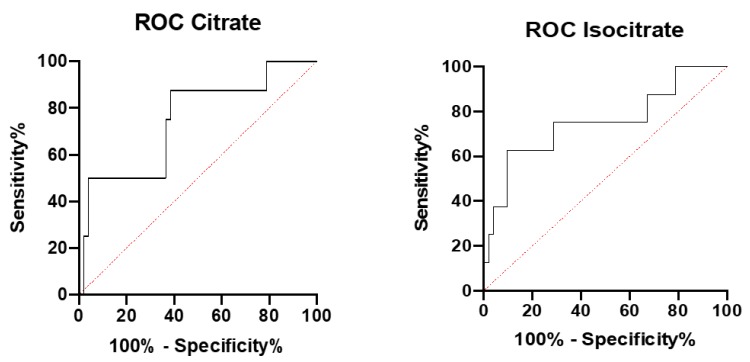
A receiver operating characteristic curve (ROC) for citrate and isocitrate by Prism 8 (GraphPad). NAFLD (*n* = 22) and controls (*n* = 67).

Diagnostic performance of isocitrate and citrate in NAFLD patients was further analyzed by establishing the receiver operating curve (ROC) and analyzing the area under the curve (AUC) (Figure 2). The accuracy of both isocitrate and citrate to discriminate NAFLD from the control cohort showed similar areas under the curve (AUROC) of 0.74 and 0.75, respectively (Table 3).

**Figure 3:**
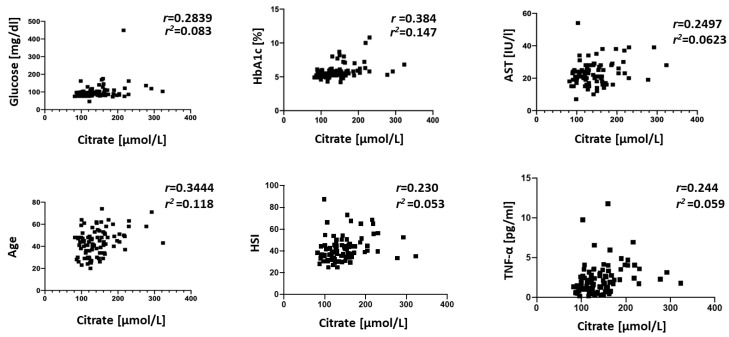

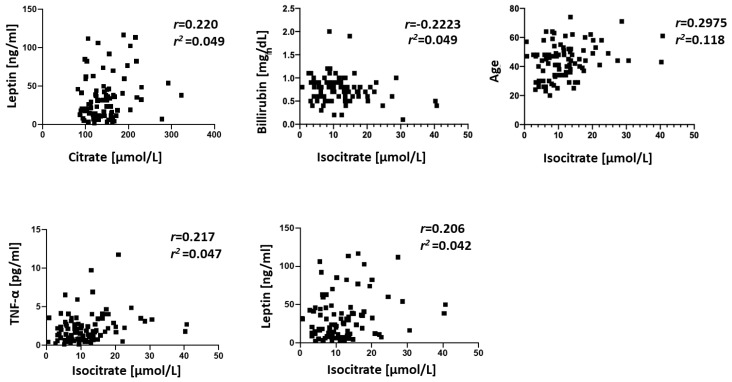
Significant correlations between isocitrate, citrate, age, HSI, and standard blood parameters for the entire cohort under the study. The strength of association between variables was calculated by Pearson’s test performed by Prism 8 (GraphPad). NAFLD (*n* = 22) and controls (*n* = 67).

Correlations between plasma Krebs cycle intermediates and standard clinical plasma metrics were explored by Pearson’s correlation coefficient. The most significant correlations were obtained in the NAFLD cohort for α-ketoglutarate to the BUN (*r* = −0.7969, *p* < 0.05). In the control group, plasma succinate was positively correlated with albumin (*r* = 0.353, *p* = 0.01), malate negatively correlated to AST (*r* = −0.332, *p* = 0.02), α-ketoglutarate was negatively correlated to glucose (*r* = −0.562, *p* < 0.001), aconitate was negatively correlated to bilirubin (*r* = −0.281, *p* = 0.04), and fumarate was negatively correlated to AST and ALT (*r* = −0.315, *p* = 0.02; *r* = −0.298, *p* = 0.03). When the entire population under the study was analyzed, the correlation analysis revealed that isocitrate was strongly correlated with age and bilirubin levels (*r* = 0.29, *p* = 0.005; *r* = −0.02, *p* = 0.04, respectively) and inflammatory markers (TNF-α and leptin), while citrate was correlated with age (*r* = 0.345, *p* < 0.001), AST activity (*r* = 0.249, *p* = 0.02), glucose levels (*r* = 0.284, *p* = 0.008), HSI, TNF-α, and leptin (Figure 3). Koliaki et al. [26] demonstrated that the metabolic flexibility of hepatic mitochondria in the early stages of NAFLD is lost at the NASH stage. We utilized calculated HSI values as a proxy of NAFLD staging and found a positive correlation to citrate levels (Figure 3).

## 4. Discussion

Nonalcoholic liver disease (NAFLD) is the most common form of chronic liver disease. There is emerging evidence that mitochondrial dysfunction contributes to NAFLD pathogenesis. However, there are no noninvasive markers that assess mitochondrial involvement in NAFLD. In this study, we analyzed plasma levels of seven Krebs cycle intermediates and investigated their correlation with the standard indices and their potential role as biomarkers of mitochondrial dysfunction in NAFLD.

As expected in NAFLD, transaminase activities were elevated (Table 2) and albumin levels were decreased. Interestingly, we did not find a significant difference in plasma creatinine levels, suggesting that the NAFLD cohort at the time of the study did not have significant renal dysfunction. No statistical difference was also found in BUN, a suggestive cardiovascular disease (CVD) risk factor in NAFLD [27]. Sixty-three percent (12/22) of NAFLD patients had BMI > 30 kg/m^2^. About thirty-two percent of the NAFLD cohort demonstrated HbA1c levels in diabetic range (>6.5%) and the same percentage of the cohort had high triglyceride levels (>200 mg/dL), indicating the presence of metabolic syndrome. An increase in inflammatory markers TNF-α and leptin are associated with NAFLD and NASH [8,28,29]. Uygun et al. [30] and Chitturi et al. [31] demonstrated in their studies that an increase in serum leptin in patients with steatohepatitis has no relation to BMI and thus can’t be justified by type 2 diabetes, sex, or obesity. In another study, Serin et al. [32] showed that elevated serum leptin is characteristic of the steatotic patients with normal transaminase levels. 

Krebs cycle has a central role in liver metabolism, connecting hepatic fatty acids and glucose-related pathways. Abnormal cellular [20] and circulating [21] Krebs cycle intermediates are closely associated with mitochondrial dysfunction, as was demonstrated in a number of studies [22,23,24,25]. Given the decreased activity in respiratory chain complexes in NAFLD [4], perturbations in cellular concentrations of Krebs cycle intermediates are expected. The data obtained for plasma Krebs cycle intermediates suggest pathophysiological insights that link mitochondrial dysfunction with NAFLD. In the present studies, NAFLD patients had higher plasma citrate levels than healthy control subjects. This finding is consistent with the van der Wier et al. study that reported elevated plasma citrate levels in NAFLD patients [33]. The study also suggested that fatty acids accumulation lead to the plasma citrate elevation in NAFLD. The increase in plasma citrate can also be associated with upregulated de novo lipogenesis in NAFLD [34,35]. Citrate serves a carbon precursor in fatty acids’ de novo biosynthesis and although less than 10% of hepatic citrate originates from plasma citrate [36], overall about 26.1% of accumulated liver triacylglycerols originate from the de novo lipogenesis [35].

We also observed an increase in circulating isocitrate levels (*p* < 0.05). Isocitrate is converted to α-ketoglutarate through oxidative decarboxylation and catalyzed by isocitrate dehydrogenase (IDH). There are three different human IDH isoforms described: NAD^+^-dependent IDH3 and NADP^+^-dependent forms IDH1/IDH2. IDH1 is highly expressed in the liver and IDH2 plays a key role in Krebs cycle regulation in multiple tissues. IDH3 is located in the mitochondria and catalyzes the rate-limiting step of irreversible conversion of isocitrate to α-ketoglutarate. Although we did not observe abnormalities in plasma α-ketoglutarate, the increase in isocitrate levels is consistent with the observation that IDH2 expression in primary hepatocytes is downregulated under palmitate stimulation and lipid accumulation [37].

For the correlation analyses, we selected all samples regardless of glucose levels (Table 1). The most prominent finding in the NAFLD cohort was a negative correlation between plasma α-ketoglutarate levels and BUN (*r* = −0.7969, *p* < 0.05). This may be related to cataplerosis (loss of Krebs cycle intermediates) of α-ketoglutarate due to hyperammonemia that has been reported in preclinical models of NAFLD [38]. This is consistent with our previous report that Krebs cycle intermediate levels, including α-ketoglutarate, were significantly lower in C2C12 myotube cells during hyperammonemia [39]. Batshaw et al. also reported a similar linear inverse correlation between plasma ammonia and α-ketoglutarate levels [40] in infants with ornithine transcarbamylase deficiency.

The correlation analysis of the entire population under the study revealed that citrate and isocitrate were correlated with age (range 23–67 years, Figure 3). Citrate was also positively correlated with glucose (*r* = 0.2839, *p* < 0.05) and HbA1c (*r* = 0.3844, *p* < 0.001). This is similar to a previous report by Shigiyama et al. that showed citrate levels are elevated in NAFLD patients with a high hepatic insulin sensitivity group [41]. The same study also reported an increase in succinate and aconitate, but we did not find a statistically significant association of these metabolites with glucose in our subjects. However, we noted that aconitate had a strong negative correlation to HDL-C in the control group (*r* = −0.26, *p* = 0.03, Supplemental data). We also noted a negative correlation of isocitrate to bilirubin that can be explained by bilirubin being a potent inhibitor of isocitrate dehydrogenase [42,43].

To evaluate isocitrate and citrate performance as mitochondrial dysfunction markers, receiver operating curves (ROCs) were constructed and areas under the curve were calculated. The AUC values indicate that isocitrate and citrate (AUC = 0.74 and AUC = 0.75) have a moderate biomarker performance. However, larger cohorts of control subjects and NAFLD patients can potentially improve these characteristics.

While serum liver disease markers for NAFLD have been established, the secondary noninvasive mitochondrial dysfunction markers in NAFLD are still to be investigated. Through the plasma Krebs cycle intermediates analysis, we present evidence of mitochondrial dysfunction involvement in the NAFLD phenotype. Our findings suggest that plasma isocitrate and citrate can discriminate between normal and NAFLD cohorts and can be utilized as noninvasive mitochondrial dysfunction biomarkers in NAFLD. Future mechanistic studies in NAFLD disease models and analysis of plasma Krebs cycle intermediates with large populations at different NAFLD stages are warranted.

Study limitations—since no liver biopsy has been performed, the assumption that NAFLD cohort has mitochondrial dysfunction is based on the multiple studies that demonstrated evidence of molecular, biochemical, and biophysical mitochondrial abnormalities in the NAFLD phenotype. It is important to note that control subjects under the study were of both genders, but the NAFLD cohort included only one male. Thus, our findings need to be validated with a more gender-inclusive group of NAFLD patients. In addition, results of this study need to be validated with large cohorts and against different stages of NAFLD, including nonalcoholic steatohepatitis (NASH) and advanced fibrosis.

## Figures and Tables

**Table 1:** Demographics and standard plasma metrics expressed as average ±standard deviation. ALP—alkaline phosphatase, ALT—alanine transferase, AST—aspartate transferase, BUN—blood urea nitrogen, HDL-C—high-density lipoprotein cholesterol, TG—triglycerides, HSI—hepatic steatosis index.

	Control (*n* = 67)	NAFLD (*n* = 22)	*p*-Value
Age (years)	42.25	47.3	
Male/Female	21/67	1/22	
BMI (kg/m^2^)	29.7 ± 7.5	40 ± 7.5	<0.001
Albumin (g/dL)	4.01 ± 0.22	3.84 ± 0.43	0.02
Bilirubin (mg/dL)	0.77 ± 0.24	0.72 ± 0.40	0.50
ALP(U/L)	93.6 ± 16.8	95.4 ± 16.37	0.70
ALT (IU/L)	22.12 ± 6.5	30.19 ± 11.53	0.001
AST (IU/L)	21.4 ± 4.54	28.42 ± 11.12	<0.001
AST/ALT	1.00 ± 0.21	0.94 ± 0.23	0.3
BUN (mg/dL)	14.2 ± 3.8	15 ± 6.7	0.49
Creatinine (mg/dL)	0.82 ± 0.22	0.8 ± 0.2	0.70
Glucose (mg/dL)	90.7 ± 17.45	127.8 ± 77.1	0.005
HbA1c (%)	5.5 ± 0.77	6.5 ± 1.6	0.001
Total chol (mg/dL)	201 ± 47	192.3 ± 41.04	0.40
HDL-C (mg/dL)	47 ± 9.95	48 ± 13	0.39
TG (mg/dL)	171 ± 9.2	192.2 ± 123	0.89
TG/HDL-C	3.72 ± 2.22	4.21 ± 2.22	0.38
TNF-α (pg/mL)	1.86 ± 1.62	3.63 ± 2.8	<0.001
Leptin (ng/mL)	23 ± 18.09	63.0 ± 34	<0.001
HSI	39.19 ± 8.07	50.42 ± 14.9	<0.001

**Table 2:** Demographics and standard plasma metrics expressed as average ±standard deviation only for samples with glucose cut-off ≤100 mg/dL.

	Control (*n* = 57)	NAFLD (*n* = 7)	*p* Value
Age (years)	42.1	46	
Male/Female	16/41	1/8	
BMI (kg/m^2^)	29.5 ± 7.9	32.4 ± 10.5	0.3
Albumin (g/dL)	3.98 ± 0.21	3.97 ± 0.32	0.92
Bilirubin (mg/dL)	0.77 ± 0.24	0.62 ± 0.16	0.07
ALP(U/L)	94.8 ± 16.1	95.8 ± 21.4	0.8
ALT (IU/L)	21.7 ± 5.4	29.6 ± 13.3	0.002
AST (IU/L)	21.1 ± 4.5	30.0 ± 13.0	<0.001
BUN (mg/dL)	14.03 ± 3.66	15.0 ± 5.5	0.50
Creatinine (mg/dL)	0.79 ± 0.18	0.75 ± 0.26	0.55
Glucose (mg/dL)	85.35 ± 9.38	88.88 ± 10.7	0.30
HbA1c (%)	5.45 ± 0.54	5.9 ± 0.4	0.44
Total chol (mg/dL)	199.3 ± 48	179.6 ± 26.2	0.23
HDL-C (mg/dL)	47 ± 10	47 ± 12	0.76
TG (mg/dL)	159 ± 84	150 ± 58.1	0.90
TG/HDL-C	3.4 ± 1.7	1.9 ± 0.84	0.84
TNF-α (pg/mL)	1.8 ± 1.1	3.57 ± 3.01	0.01
Leptin (ng/mL)	24.18 ± 18.6	61.68 ± 32.2	<0.001
HSI	38.9 ± 8.35	41.27 ± 11.36	0.45

**Table 3:** Evaluation of isocitrate and citrate as mitochondrial dysfunction markers in NAFLD. AUC—area under the curve.

Marker	AUC	*p*-Value	Error
Citrate	0.749	*p* < 0.05	0.097
Isocitrate	0.750	*p* < 0.05	0.102

## Supplementary Materials

The following are available online at https://www.mdpi.com/2077-0383/9/2/314/s1, Figure S1: Post derivatization structures of citric acid cycle metabolites under the study, Table S1: Target ions and retention times for all Krebs cycle intermediates and internal standards. Figure S2: Tricarballylic acid EI-MS spectrum. Figure S3 (A) isocitrate and (B) citrate EI-MS spectra. m/z 245 is specific to isocitrate, Figure S4. Calculated hepatic steatosis index (HSI).
