# Supplementary material for: Plasma Krebs Cycle Intermediates in Nonalcoholic Fatty Liver Disease
Source: J Clin Med. 2020 Jan 22;9(2):314. doi: 10.3390/jcm9020314 (PMC7073566; doi:10.3390/jcm9020314)

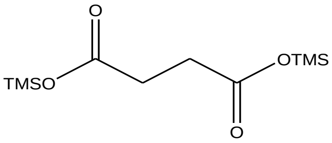

Succinate

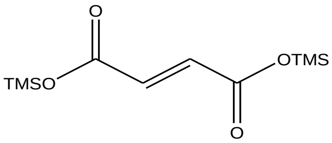

Fumarate

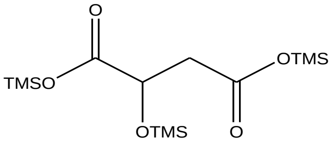

Malate

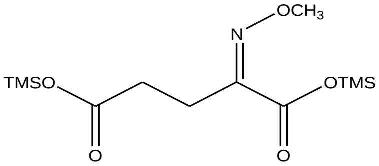

a-Ketoglutarate

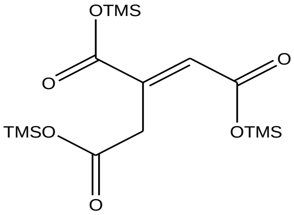

Aconitate

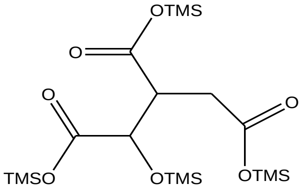

Isocitrate

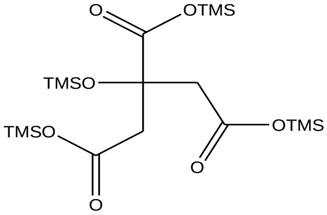

Citrate

Supplemental Figure 1: Post derivatization structures of citric acid cycle metabolites under the study. TMS- Trimethylsilyl

**Analyte/Internal standard**	**RT**	**Target ions**
Succinate/ Succinate d_6*	7.22	*m/z* 247; 251
Fumarate/ Fumarate $^{13}C_4$	7.53	*m/z* 245; 249
Malate/ Malate $^{13}C_4$	8.8	*m/z* 233; 236
α-Ketoglutarate/ α- Ketoglutarate d_6*	9.5	*m/z* 304; 308
Citrate/ Citrate $^{13}C_6$	11.4	*m/z* 273; 278
Aconitate/Tricarballylic acid	10.8/10.7	*m/z* 229; 377
Isocitrate/Tricarballylic acid	11.4/10.7	*m/z* 245; 377

*Deuterium labeling on acidic positions is lost during derivatization process.
Supplemental Table 1: Target ions and retention times for all analytes and internal standards

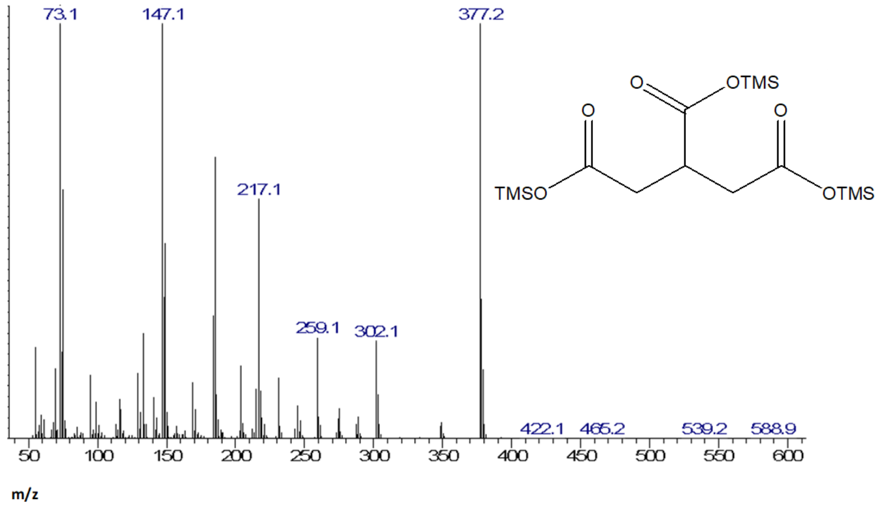

Supplemental figure 2: Tricarballylic acid EI-MS spectrum

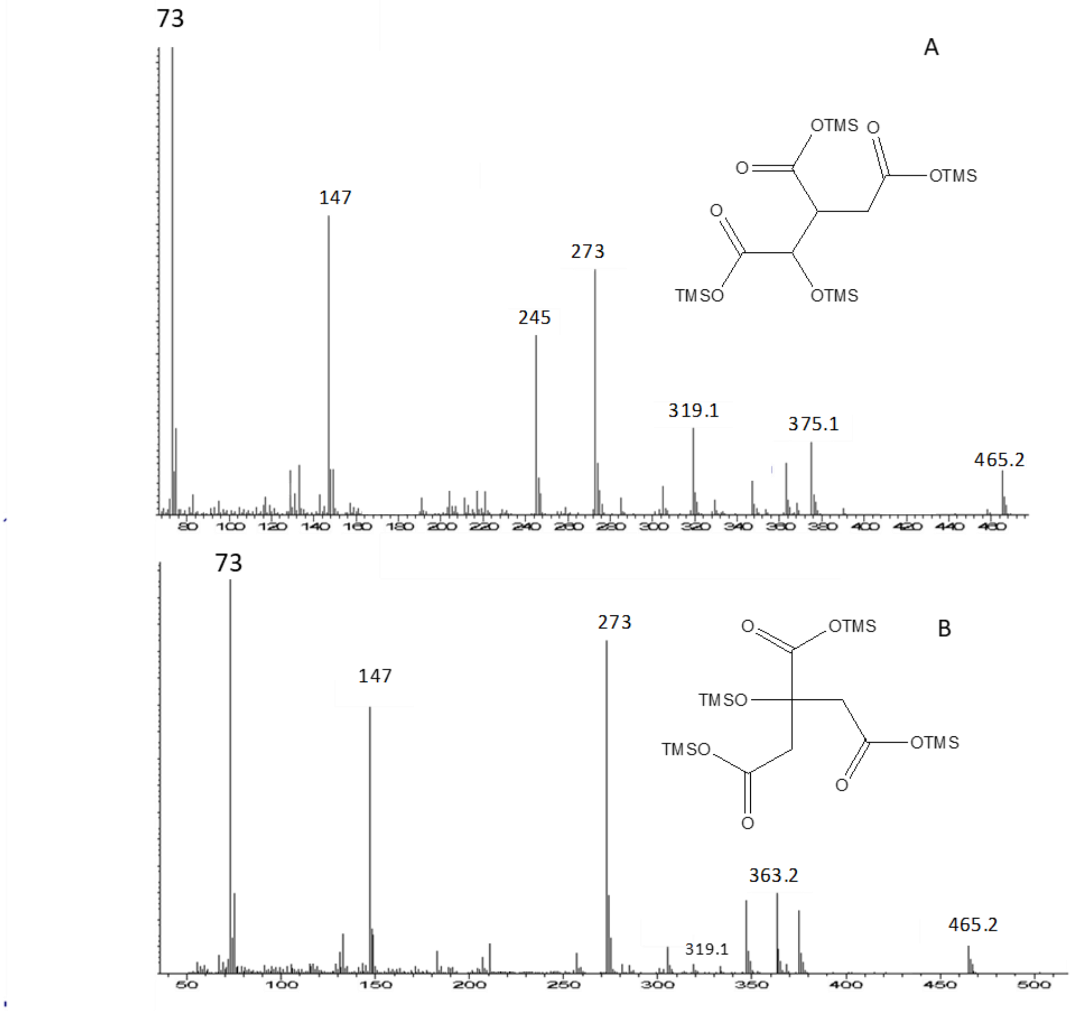

Supplemental figure 3: (A) isocitrate and (B) citrate EI-MS spectra. m/z 245 is specific to isocitrate

Supplemental Figure 4: Calculated HSI. NAFLD (n=22) and control (n=67). Statistical analysis performed by Prism 8 (GraphPad). * $p<0.05$

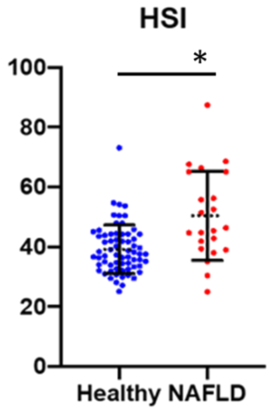

Supplement: Supplementary file 1 [file jcm-09-00314-s001.pdf]
